# Systematic Understanding of Mechanisms of a Chinese Herbal Formula in Treatment of Metabolic Syndrome by an Integrated Pharmacology Approach

**DOI:** 10.3390/ijms17122114

**Published:** 2016-12-16

**Authors:** Meimei Chen, Fafu Yang, Xuemei Yang, Xinmei Lai, Yuxing Gao

**Affiliations:** 1 College of Chemistry and Chemical Engineering, Fujian Normal University, Fuzhou 350007, China; 2 College of Traditional Chinese Medicine, Fujian University of Traditional Chinese Medicine, Fuzhou 350122, China; mei_tcm@163.com (X.Y.); keerer1990@163.com (X.L.); 3 College of Chemistry and Chemical Engineering, Xiamen University, Xiamen 361005, China; gaoxingchem@xmu.edu.cn

**Keywords:** metabolic syndrome, molecular docking, network analysis, Wendan decoction

## Abstract

Metabolic syndrome (MS) is becoming a worldwide health problem. Wendan decoction (WDD)—a famous traditional Chinese medicine formula—has been extensively employed to relieve syndromes related to MS in clinical practice in China. However, its pharmacological mechanisms still remain vague. In this study, a comprehensive approach that integrated chemomics, principal component analysis, molecular docking simulation, and network analysis was established to elucidate the multi-component and multi-target mechanism of action of WDD in treatment of MS. The compounds in WDD were found to possess chemical diversity, complexity and drug-likeness compared to MS drugs. Six nuclear receptors were obtained to have strong binding affinity with 217 compounds of five herbs in WDD. The importance roles of targets and herbs were also identified due to network parameters. Five compounds from *Radix Glycyrrhizae Preparata* can hit all six targets, which can assist in screening new MS drugs. The pathway network analysis demonstrated that the main pharmacological effects of WDD might lie in maintaining lipid and glucose metabolisms and anticancer activities as well as immunomodulatory and hepatoprotective effects. This study provided a comprehensive system approach for understanding the multi-component, multi-target and multi-pathway mechanisms of WDD during the treatment of MS.

## 1. Introduction

Metabolic syndrome (MS) is a complex syndrome cluster of metabolic disturbances, including typically obesity, insulin resistance, dysglycemia, dyslipidemia and hypertension, at increase risk of type 2 diabetes mellitus, atherosclerosis and cardiovascular events [1]. Nowadays, prevalence of MS is increasing significantly, which has been recognized as a worldwide health problem [2,3]. The crucial roles of insulin resistance and energy imbalance are commonly considered as the main causes of MS [4]. Although the roles of the two main causes are plausible, there is still no single treatment to control MS. Current therapeutic strategies for this disease are still aimed at to treat each component separately [5].

Traditional Chinese medicine (TCM), as an important complementary and alternative medical system, has been practiced in China for thousands of years [6,7]. Herbal medicine therapy has been an alternative and promising strategy for the treatment of MS in China [8]. A well-known herbal formula WDD, developed in the Tang Dynasty, has been extensively employed in recovery of complex diseases including syndromes related to MS in clinical practice in China [8,9,10]. WDD had improved clinical therapeutic effects for eliminating phlegm, anti-inflammation, lowering blood pressure, glucose and cholesterol levels, and reducing weight and waistline [11,12]. A meta-analysis of comparing treatment efficacy for MS patients using the WDD versus western conventional therapeutics, involving 2512 patients and 1282 participants in the intervention groups, found that the overall efficacy rate was 91.4% for WDD and 66.9% for the control treatments, and also confirmed that adverse events were rare and minor [8]. This formula is comprised of *Radix Glycyrrhizae Preparata*, *Citrus Aurantium*, *Pericarpium Citri Reticulatae*, *Poria Cocos*, *Pinellia Ternata* and *Caulis Bambusae* in *Taeniam*, all of which are officially recorded in the Chinese Pharmacopoeia [13]. The pharmacological effects of these six herbs are diverse and complex, which contribute to the recovery and protection of the nervous, respiratory, digestive, urinary, immune, cardiovascular and endocrine systems [13]. For example, *Radix Glycyrrhizae Preparata* is one of the oldest and most frequently used herbs in TCM and exhibits a variety of biological activities, including antitumor, antimicrobial, anti-inflammatory, antiviral, immunoregulatory, inhibitory effects on diabetes, hepatoprotective effects, enhancing memory and nerve protective effects, an estrogen-like effect, antiangiogenic property, and reducing pains [14]. Thus, WDD, as the combination formula of these six herbs, the pharmacological mechanisms are more elusive. To date, the active chemical ingredients and action mechanisms still remain vague for this formula in treatment of MS. Considering the fact that herbal formulas are mixtures of hundreds of chemical compounds and act on multiple cellular targets, it is difficult to systematically study the mechanisms of herbal formulas using routine methods [7]. Therefore, the identification of complex molecular mechanisms is a major challenge in herbal formulas research. Thus, new methods and strategies such as network pharmacology are urgently needed to address this problem. Network pharmacology, proposed by Andrew L. Hopkins, clarifies the synergistic effects and the underlying mechanisms of multi-component and multi-target agents by analyzing various networks of complex and multilevel interactions [15,16]. This method has been widely applied in systematic understanding of the mechanisms of herbal formula [7,17,18]. For example, it was used to identify 1327 targets of 673 compounds from nine herbs involved in Fc epsilon RI signaling pathway and regulation of immunoglobulin production from a Chinese herb formula on rheumatoid arthritis [7]. Gao et al. ascertained molecular targets of herbs prolonging survival time of patients with advanced hepatocellular carcinoma (HCC) based on this method [18].

In this study, we have developed a comprehensive systems approach to investigate the pharmacological mechanisms of WDD acting on MS. First, we analyzed the chemical group composition of the ingredients in WDD, explored their chemical characteristics and distribution in chemical space, and measured their drug-likeness properties compared to drugs related with MS (MS drugs) approved by Food and Drug Administration (FDA); Second, the biological targets and active ingredients of WDD were screened by molecular docking. The compound with docking score higher than the original ligand in the crystal structure of protein-ligand complex was believed to have a strong affinity with the target [19]; Third, an investigation into the relationships among active compounds of WDD, herbs, potential targets and their related signaling pathways was analyzed using network analysis and Kyoto Encyclopedia of Genes and Genomes (KEGG) [20], respectively, which offers a great opportunity for a deep understanding of the pharmacological mechanisms of WDD.

## 2. Results and Discussion

### 2.1. Chemical Diversity and Drug-Likeness Analysis of Wendan Decoction (WDD)

To give an overview of the ingredient families in WDD, chemical ingredients in WDD were classified into five general categories: phenylpropanoids, flavonoids, alkaloids, sugars and terpenoids, which can be subdivided into 17 subclasses, listed in Table 1. The number of different categories was varied in order: flavonoids > sugars > terpenoids > phenylpropanoids > alkaloids. The main ingredients of *Radix Glycyrrhizae Preparata* were flavonoids, terpenoids, phenylpropanoids and alkaloids, such as glycyrol, glycyrrhizin, glycyrin and liconeolignan. *Citrus Aurantium* mainly included flavonoids, alkaloids and sugars, such as tangeritin, synephrine and dehydrodiconiferyl alcohol-4-β-d-glucoside. *Pericarpium Citri Reticulatae* mainly contained terpenoids and flavonoids, such as limonene, hesperidin, tangeretin. The main ingredients of *Poria Cocos* were terpenoids and sugars, such as pachymic acid, tumulosic acid, pachy-man and pachymaran. *Pinellia Ternata* mainly contained terpenoids and sugars, such as 1,2,3,4,6-penta-ogalloylglucose. *Caulis Bambusae* in *Taeniam* mainly consist of phenylpropanoids such as coniferyl aldehyde [13]. From the total amount of compounds in Table 1, we can conclude that some compounds simultaneously had some different structural features, which belong to different classifications. These results showed that the ingredients from WDD possessed chemical diversity and complexity.

**Figure 1:**
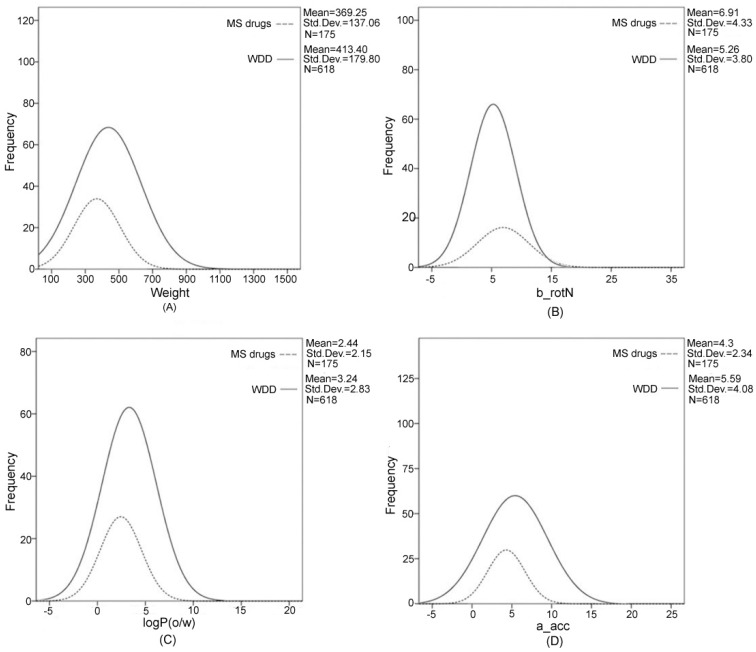

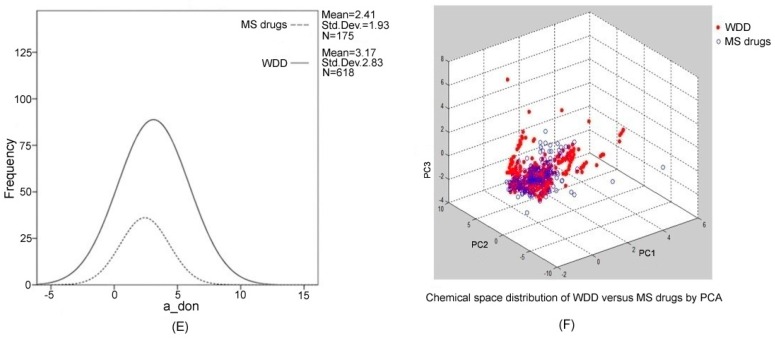
Comparing chemical characteristics of ingredients in WDD versus metabolic syndrome (MS) drugs. (**A**–**E**) are distributions of drug-like properties of ingredients in WDD and MS drugs; (**F**) chemical space distribution of WDD versus MS drugs by principal component analysis (PCA).

Additionally, the drug-likeness analysis was also performed to evaluate the oral drug-like property of chemical ingredients in WDD using the Lipinski rule of five, that is, molecular weight lower than 500 Da, number of H-bond donors (a don) less than five, number of H-bond acceptors (a_acc) less than 10, number of rotatable bonds (b_rotN) lower than 10 and octanol-water partition coefficient (logP(*o*/*w*)) lower than five, is of importance for the screening of drugs with pharmacological activity [21,22]. The drug-like property descriptors of WDD ingredients were listed in Table 2. All mean and median values of five such descriptors were coincided with the five rules of oral medications. Among them, 75.1% of the compounds had a molecular weight <500, 87.1% had <10 H-bond acceptors, 83.2% had less than 5 H-bond donors, 78.3% had a LogP of less than five, and 92.9% had <10 b_rotN, respectively. Next, to provide insight into the absorption, distribution, metabolism, excretion and toxicity (ADMET) properties of these ingredients, these five drug-like properties of the ingredients in WDD were compared with synthesis drugs concerning MS diseases approved by FDA (Figure 1A–E) [22,23,24]. The overall shapes of the distributions of these characteristics were similar between the ingredients in WDD and MS related drugs, but the standard deviations for ingredients in WDD were larger than for MS drugs except b_rotN. This result indicated that the weight, H-bond acceptors, H-bond donors and water solubility of ingredients in WDD were more variable than those of MS drugs. The proportion of compounds with more than 10 rotatable bonds (b_rotN) in WDD exceeded MS drugs, which meant the structures of ingredients in WDD were more flexible. Thus, it can be concluded that the ingredients in WDD possessed chemical diversity and drug-likeness compared to MS drugs approved by FDA.

Further, to comprehensively investigate the chemical property differences between ingredients in WDD and MS drugs, we reported herein a principal component analysis (PCA) of the 30 common structural and physicochemical features [24]. Figure 1 plotted the chemical space distribution of the ingredients in WDD and MS drugs. The three principal components (PC1, PC2 and PC3) can explain almost 90% of the variance in 30 physicochemical characteristics of WDD ingredients and MS drugs with eigenvalues bigger than two, respectively, indicating that they can be used to analyze the chemical space distribution. As seen in Figure 1, the chemical space distribution of WDD had a considerable overlap but larger than that of MS drugs, indicating that they had great similarity, but also maintained their own characteristics. Herein, it can be concluded that WDD ingredients exhibited greater chemical diversity and greater three-dimensional complexity compared to MS drugs.

### 2.2. Potential Targetsand Bioactive Compounds of WDD

The putative targets of the WDD compounds were performed by the molecular docking method, which has been successful in prioritizing large chemical libraries to identify experimentally active compounds [25]. A total of 217 compounds were found to be well interacted with six targets, with higher dock scores than the cut-off values. On average, each target had 36 putative compounds. Detailed information of docking results was listed in Table 3. These bioactive compounds derived from five herbs, including *Radix Glycyrrhizae Preparata*, *Poria Cocos*, *Pericarpium Citri Reticulatae*, *Pinellia Ternate* and *Citrus Aurantium*. The number of bioactive compounds per herb in Table 3 suggested the crucial role of *Radix Glycyrrhizae Preparata* in WDD. As shown in Table 3, all herbs simultaneously shared five common targets, suggesting the synergistic and multi-target effects of WDD in treatment of MS. Of note, *Radix Glycyrrhizae Preparata* can interact with all six targets. These targets consisted of peroxisome proliferators-activated receptors (PPARs: PPARα, PPARβ and PPARγ), liver X receptors (LXRs: LXRα and LXRβ) and retinoid X receptor α (RXRα), all of which are members of the nuclear hormone receptor superfamily. PPARs regulate the expression of a group of genes that maintain glucose and lipid metabolism, including inhibition of lipid deposition, improving insulin sensitivity, promoting adipocyte differentiation and activating the fatty acids store in adipocyte, reducing the serum levels of fatty acids, which are closely associated with the development of MS [26]. LXRs are glucose sensors, which are involved in the regulation of cholesterol, lipid metabolism and liver carbohydrate metabolism [27]. RXRα regulates multiple metabolic pathways in modulating cholesterol, fatty acid, bile acid, steroid and xenobiotic metabolism and homeostasis in liver and modulates adipogenesis and lipolysis in adipocytes. RXRα agonists had also been shown to reduce hyperglycemia, hypertriglyceridemia and hyperinsulinemia [28]. The biological functions of these six targets are closely associated with the mechanism of MS. In addition, PPARα had been reported to be influenced by WDD in the treatment of MS rats [29]. Herein, it can be concluded that these six targets played a key role in WDD treating MS.

**Figure 2:**
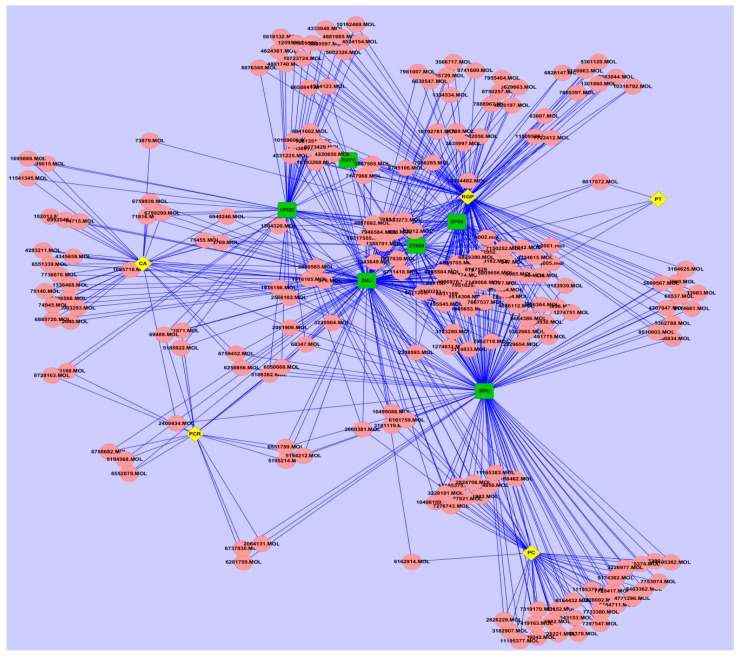
The herb-compound-target network: the pink round nodes refer to compounds from herbs; the yellow rhombic nodes represent herbs; the green quadrate nodes represent targets.

Further to illuminate the relationship between bioactive compounds and hit targets, the herb-compound-target network was constructed. As shown in Figure 2, the herb-compound-target network contained 228 nodes (217 compounds, six potential targets and five herbs). The network centralization and network heterogeneity were 0.618 and 2.732, respectively, indicating that a few nodes were more central than others in the network. Table 3 listed a few simple parameters of the H-C-T network such as degree and betweenness, which have been proposed as metrics in assessing major nodes. Generally, the larger a node’s degree or betweenness is, the more important the node is in the interaction network [30]. As seen in Table 4, PPARγ had the largest degree (145) and betweenness (0.3741), indicating that PPARγ was a crucial target involved in the treatment of MS by WDD. Correctively, PPARγ is abundantly expressed in adipose tissue and is primarily involved in the regulation of lipid and glucose metabolisms, which seems to be related with every aspect of MS [31]. For instance, PPARγ is a molecular target for insulin-sensitizing thiazolidinedione drugs such as troglitazone, pioglitazone, and rosiglitazone, which have been approved to enhance insulin secretion and improve glucose tolerance for use in the treatment of type 2 diabetes patients [32]. Thereby, according to the different degrees of targets in the network, the importance of these targets involved in treatment of MS by WDD was varied in order: PPARγ > LXRα > PPARα > LXRβ > PPARβ > RXRα. Similarly, it can also be deduced that the importance of these herbs in treatment of MS was varied in order: *Radix Glycyrrhizae Preparata* > *Poria Cocos* > *Citrus Aurantium* > *Pericarpium Citri Reticulatae* > *Pinellia Ternate*. *Radix Glycyrrhizae Preparata* possessed more than a half of the bioactive ingredients of WDD, which can interact with all six targets. It has been proved to possess a wide range of pharmacological effects, which may contribute to the anti-diabetes, anti-abdominal obesity, antiobesity effects and anticancer activities [33]. For instance, Kuroda et al. reported that an EtOH extract of *Glycyrrhiza Uralensis F. Roots*, was effective in preventing and/or ameliorating diabetes, abdominal obesity, and hypertension in KK-Ay mice, high-fat diet-induced obese C57BL/6J mice, and spontaneously hypertensive rats, respectively [34]. Thus, it can be seen that the network analysis results further confirmed the accuracy of docking results. Although it has been reported that large doses or long-term injections of licorice alone sometimes produce sodium retention and hypokalemia [14]. Formulas containing licorice were proved to not significantly influence the potassium levels in routine clinical herbal therapies [14].

**Figure 3:**
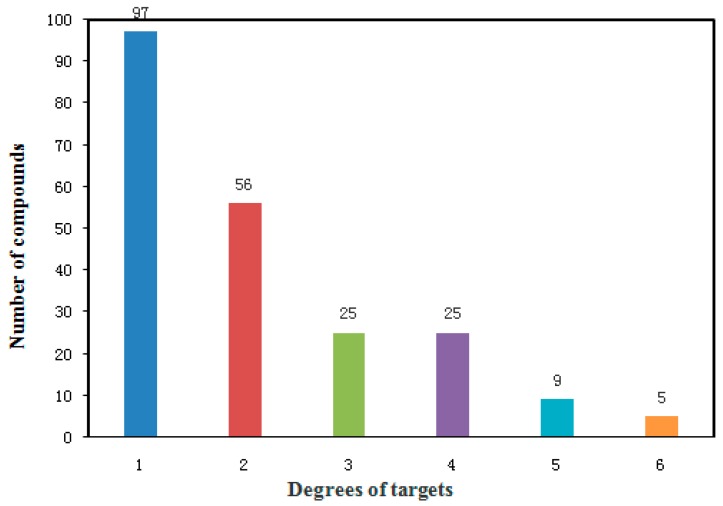
Degree distribution between compounds and targets.

The degree of distribution between compounds and targets was also presented in Figure 3. It can be seen that 120 potential compounds had more than two links with other targets. Among them, five compounds had high-degree distributions, and each of them hit six targets. Their chemical names and bioactivity are listed in Table 5. They all came from *Radix Glycyrrhizae Preparata*, such as glabrol, euchrestaflavanone A, euchrenone A5 and glyinflanin D. In general, the compounds with higher degree of connectivity are more potent pharmacologically. For instance, glabrol possessed a wide range of pharmacological effects, including activation of PPARγ and D(3) dopamine receptor isoform e (Drd3), and inhibition of Tyrosine-protein phosphatase non-receptor type 1(TPNT1) and Protein-tyrosine phosphatase 1B (PTP1B) [35]. Euchrestaflavanone A was found to have beneficial biological effects, including cytotoxic activity, inhibition of TPNT1 and PTP1B, and antimicrobial activity [36]. Overall, these five compounds can assist in screening new MS drugs.

Further, to analyze compounds with good pharmacokinetic properties among 217 bioactive compounds, the pharmacokinetic parameters including human intestinal absorption and aqueous solubility were calculated by using DS 2.0. In general, compounds with human intestinal absorption levels of 0–2 and aqueous solubility levels of 2–4 were considered with good pharmacokinetic properties [37,38]. After the pharmacokinetic parameters calculations, 110 compounds were predicted to have good absorption and aqueous solubility, which can be considered as lead drugs in the development of new drugs concerning MS. They can be seen in the supplementary file (Table S1).

### 2.3. The Potential Pathways Affected by WDD

**Figure 4:**
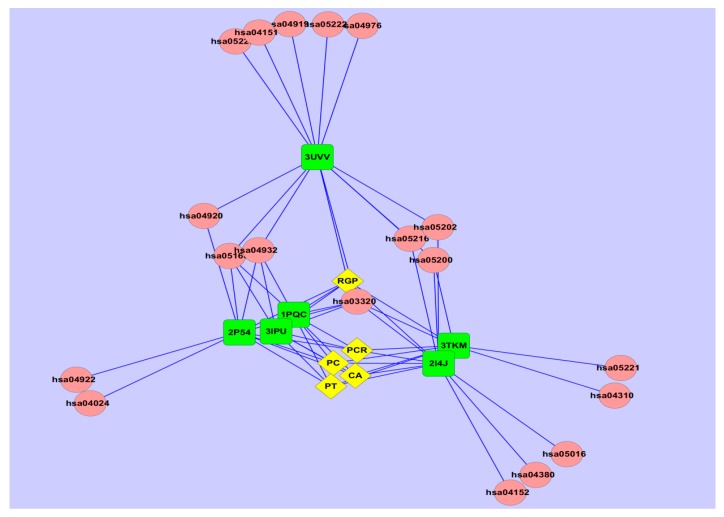
The herb-target-pathway network: the pink round nodes referred to pathways; the yellow rhombic nodes represent herbs; the green quadrate nodes represent targets.

Finally, to explore potential biological pathways that WDD might impact in the treatment of MS, the herb-target-pathway network was constructed. As shown in Figure 4, 19 pathways were identified and listed in Table 6. The results contained endocrine-related pathways such as the PPAR signaling pathway, the Thyroid hormone signaling pathway, the Adipocytokine signaling pathway, the Glucagon signaling pathway and Non-alcoholic fatty liver disease (NAFLD); signal transduction-related pathways such as the PI3K-Akt signaling pathway, the AMPK signaling pathway, the cAMP signaling pathway and the Wnt signaling pathway; and cancer-related pathways such as Pathways in cancer, Thyroid cancer, Transcriptional misregulation in cancer, Small cell lung cancer and Non-small cell lung cancer, and infectious liver diseases such as Hepatitis C. These pathways were mainly involved in lipid and glucose metabolisms, cancers and liver diseases. As is commonly known, the unbalance of lipid and glucose metabolisms are the main causes of MS; our results can further illuminate the mechanism of MS. As shown in Table 6, the PPAR signaling pathway has the largest degree, in which all the targets participate. The result suggested that it was a crucial pathway of WDD in the treatment of MS. In fact, the PPAR signaling pathway was frequently involved in various biological processes, including controlling lipid metabolism, cell proliferation and blood glucose uptake, which are particularly critical for MS. Additionally, experimental reports had proved that lipid and glucose metabolic-related pathways such as PPAR, AMPK and PI3K-Akt were regulated by WDD in the treatment of MS [39,40,41]. Therefore, it can be concluded that WDD treated MS by affecting lipid and glucose metabolisms, and the above 19 pathways can be worthwhile for further explored, especially for some pathways with high degrees, i.e., NAFLD, Hepatitis C and Pathways in cancer. NAFLD is considered as the hepatic manifestation of MS. Growing clinical studies have proved that WDD can treat NAFLD involved in improving syndromes, reducing serum enzymes of liver function, lowering the blood lipids and removing hepatic steatosis [42]. However, no studies have assessed the influence of WDD in the regulation of Hepatitis C and Pathways in cancer. But, some herbs in WDD were reported to have liver protection and anti-cancer effects, respectively. For instance, chemical ingredient of licorice (glycyrrhizin) has been widely used in the treatment of a variety of liver diseases, such as cirrhosis of the liver, hepatitis B, and hepatitis C [14]. Sun et al. reported that roasted licorice extract reduced the viability of breast cancer cells and blocked cancer cell-mediated expression of receptor activator of NF-κB ligand in osteoblasts, as well as inhibiting RANKL-induced osteo-clastogenesisin bone marrow-derived macrophages [43]. Therefore, the regulatory role of WDD in the above 19 pathways might provide some new insight into the underlying molecular basis and therapeutic effects of WDD, especially in endocrine system and cancers.

## 3. Materials and Methods

### 3.1. Chemical Structures Collection and Structural Classification

Chemical ingredients from herbs in WDD were collected from the Beilstein/Gmelin CrossFire Chemical database, Chinese Herbal Drug Database (2002 version) and the Handbook of the Constituents in Chinese Herb Original Plants [44,45]. In total, we collected the structural information of 282 compounds for *Radix Glycyrrhizae Preparata*, 156 compounds for *Citrus Aurantium*, 110 compounds for *Pericarpium Citri Reticulatae*, 54 compounds for *Poria Cocos*, 35 compounds for *Pinellia Ternata* and 5 compounds for *Caulis Bambusae* in *Taeniam*. After removing duplicates, 618 compounds were retained and their 2D structures were sketched using ISIS Draw 2.5 (Molecular Design Limited (MDL) Information Systems, Inc., San Leandro, CA, USA), and then they were grouped based on the criteria for chemical structure classification of natural products. Their 3D structures were further optimized using MOE2008 (Chemical Computing Group, Montre, Montreal, QC, Canada) with a Merck molecular force field (Merck Research Laboratories, Boston, MA, USA). In addition, a total of 175 synthesis drugs concerning MS diseases such as diabetes, hypertension, hyperlipidemia and obesity approved by Food and Drug Administration were collected from the DrugBank database [46] and their structures were optimized using the same method with herb chemical ingredients (Table S2).

### 3.2. Chemical Space and Drug-Likeness Calculations

The molecular structures are directly related with their physicochemical properties. Molecular descriptors are characterization parameters of chemical structures. Thus, molecular descriptors can be calculated to analyze the chemical space information and the drug-likeness. In this study, the optimized structures were used to calculate 30 common descriptors by employing the QuaSAR module of MOE. These calculated descriptors include three classes: 2D descriptors such as Weight, logP(*o*/*w*) and number of rings, which use the atoms and connection information of the molecules, internal 3D (i3D) descriptors such as dipole, ASA and VSA, which use 3D coordinate information about each molecule and external 3D (x3D) descriptors such as, which uses 3D coordinate information with an absolute frame of reference [47]. Then, the principal component analysis was applied to analyze chemical ingredients distribution in the chemical space [48]. In addition, the Lipinski’s rule of five was used to evaluate the drug-likeness properties descriptors of the herbal compounds [49].

### 3.3. Target Prediction and Bioactive Compound Screening for WDD

In this study, molecular docking was employed to calculate the binding efficacy of compounds in WDD and targets. The crystal structures of the protein-ligand complexes of 9 protein targets closely associated with MS reported by literatures [50], including peroxisome proliferators-activated receptors (PPARα, PPARβ, PPARγ), liver X receptors (LXRα, LXRβ), retinoid X receptor (RXRα, RXRβ, RXRγ) and farnesoid X recept (FXR) were retrieved from the Research Collaboratory for Structural Bioinformatics Protein Data Bank (RCSB PDB; www.rcsb.org). Crystallographic water molecules were removed and hydrogen atoms were added in the file. After that, the compounds from herbs in WDD were docked to these targets using the autodock 4.0 program. For each docking, the binding site was set as a 40 × 40 × 40 Å cube centered on the occupied space of the original ligand and the spacing of energy grid points was defined as 0.375’ [18]. Default settings were used for all the other parameters. The compounds that docking scores were higher than the original ligands in the crystal structures of the protein-ligand complexes were believed to have a strong affinity with the targets [19]. So the targets and compounds with the higher dock scores than those of the original ligands were selected as potential targets and bioactive compounds.

### 3.4. Network Construction and Analysis

The analysis of the targets, herbs, compounds and biological pathways may provide a practical therapeutic strategy against the disease. In this study, the screened targets and bioactive compounds together with corresponding herbs were firstly utilized for herb-compound-target network construction. We also performed a pathway enrichment analysis using pathway data obtained from the KEGG Database [20]. Then, the herb-target-pathways network was established by using screened targets, their corresponding pathways and herbs. These two networks were constructed by Cytoscape 2.8.3 and analyzed by the network analysis plugin [51].

## 4. Conclusions

In this paper, a comprehensive approach that integrated chemomics, principal component analysis, molecular docking simulation and network analysis was established to elucidate the multi-component and multi-target mechanism of action of WDD in treatment of MS. Our main findings were as follows: First, the ingredients in WDD possessed chemical diversity, complexity and drug-likeness compared to MS related drugs approved by FDA. Second, six targets including PPARα, PPARβ, PPARγ, LXRα, LXRβ and RXRα were predicted to have strong binding affinities with 217 active ingredients of five herbs in WDD. The biological functions of these six targets are closely associated with the mechanism of MS. Additionally, PPARα had been reported to be influenced by WDD in the treatment of MS rats. Thus, this result can provide clues to investigate the pharmacological mechanisms of WDD for the treatment of MS. Third, the herb-compound-target network of WDD provided insights into the synergetic effects among the herbs contained in this formula. According to the network parameters, the importance of targets and herbs in treatment of MS by WDD were identified in order: PPARγ > LXRα > PPARα > LXRβ > PPARβ > RXRα and *Radix Glycyrrhizae Preparata* > *PoriaCocos* > *Citrus Aurantium* > *Pericarpium Citri Reticulatae* > *Pinellia Ternate*, respectively. Five compounds from *Radix Glycyrrhizae Preparata* can affect all six predicted targets. Four of them were reported to have pharmacological properties such as antiinflammatory, antiviral, antimicrobial, antidiabetic, and anticancer activities. Therefore, these compounds possessed diverse biological activity, which can assist in screening new MS drugs. Among 217 bioactive compounds, 110 compounds were predicted to have good absorption and aqueous solubility, which can be considered as lead drugs in the development of new drugs concerning MS. Fourth, the pathway network analysis showed that 19 pathways were affected by WDD during the treatment of MS and the key pharmacological effects and therapeutic indications of WDD might lie in maintaining lipid and glucose metabolisms and anticancer activities as well as immunomodulatory and hepatoprotective effects. Several signaling pathways, i.e., the PPAR signaling pathway, AMPK signaling pathway and PI3K-Akt signaling pathway, had been reported to be impacted by WDD during the treatment of MS. Therefore, the pathway data might provide some new insight into the underlying molecular basis and therapeutic effects of WDD, especially in the endocrine system and cancers, which can help find new therapeutic effects of WDD and optimize clinical usage of this formula. Overall, this study provides an effective and accurate strategy for understanding the multi-target and multi-component mechanism of WDD during the treatment of MS in a holistic way.

## Figures and Tables

**Table 1:** Structural categories of ingredients in Wendan decoction (WDD).

Subclasses	Number of Ingredients	Categories
Lignin	1	Phenylpropanoids
Coumarin	47	Phenylpropanoids
Flavan-3-ol	1	Flavonoids
Isoflavanone	10	Flavonoids
Anthocyanidin	8	Flavonoids
Chalcone	16	Flavonoids
Flavonol	19	Flavonoids
Isoflavone	44	Flavonoids
Flavone	63	Flavonoids
Flavanone	53	Flavonoids
Dihydrochalcone	69	Flavonoids
Imidazole	1	Alkaloids
Piperidines	4	Alkaloids
Quinolines	4	Alkaloids
Indoles	5	Alkaloids
Pyrrolidines	12	Alkaloids
Six carbon aldose	99	Sugars
Five carbon aldose	103	Sugars
Annular monoterpene	90	Terpenoids
Open chain monoterpene	10	Terpenoids
Diterpene	96	Terpenoids

**Table 2:** Drug-like property descriptors of compounds in WDD.

Descriptors	Meaning	Median	Mean	Std. Deviation
Weight	Molecular weight	388.47	413.40	179.80
a_acc	Number of hydrogen bond acceptor atoms	5	5.59	4.08
a_don	Number of hydrogen bond donor atoms	2	3.17	2.83
b_rotN	Number of rotatable bonds	5	5.26	3.80
logP(*o*/*w*)	Log of the octanol/water partition coefficient	3.28	3.24	2.83

**Table 3:** Docking results of WDD.

Herbs	Hit Targets	Number of Bioactive Compounds
*Radix Glycyrrhizae Preparata*	PPARα, PPARβ, PPARγ, LXRα, LXRβ, RXRα	118
*Poria Cocos*	PPARα, PPARβ, PPARγ, LXRα, LXRβ	42
*Citrus Aurantium*	PPARα, PPARβ, PPARγ, LXRα, LXRβ	35
*Pericarpium CitriReticulatae*	PPARα, PPARβ, PPARγ, LXRα, LXRβ	18
*Pinellia Ternata*	PPARα, PPARβ, PPARγ, LXRα, LXRβ	3

**Table 4:** Network features of targets and herbs in the herb-compound-target network.

Code	Node	Degree	Betweenness
2P54	PPARα	67	0.0537
2I4J	PPARγ	145	0.3741
3UVV	RXRα	9	0.0007
3IKM	PPARΔ	48	0.0235
3IPU	LXRα	128	0.3283
1PQC	LXRβ	62	0.0921
RGP	*Radix Glycyrrhizae Preparata*	118	0.2311
PC	*Poria Cocos*	42	0.0264
CA	*Citrus Aurantium*	35	0.0232
PCR	*Pericarpium Citri Reticulatae*	18	0.0074
PT	*Pinellia Ternata*	3	0.0001

**Table 5:** Five compounds with the highest degree distribution between compounds and targets.

Chemical Name	Herb Source	Degree	Bioactivity
Glabrol	Radix Glycyrrhizae Preparata	6	Activation of PPARγ and Drd3, and inhibition of TPNT1 and PTP1B.
Euchrestaflavanone A	Radix Glycyrrhizae Preparata	6	Inhibition of TPNT1 and PTP1B, and cytotoxic activity, antimicrobial activity
Euchrenone a5	Radix Glycyrrhizae Preparata	6	Activation of PPARγ
Glyinflanin D	Radix Glycyrrhizae Preparata	6	Unreported
1-(7-Hydroxy-2,2-dimethyl-2*H*-chromen-6-yl)-3-(4-hydroxy-3-(3-methylbut-2-en-1-yl)phenyl)propane-1,3-dione	Radix Glycyrrhizae Preparata	6	Cytotoxic activity

**Table 6:** 19 Kyoto Encyclopedia of Genes and Genomes KEGG pathways associated with 6 predicted targets of WDD.

Pathway ID	Term	Pathway Class	Degree
hsa03320	PPAR signaling pathway	Endocrine system	6
hsa04919	Thyroid hormone signaling pathway	Endocrine system	1
hsa04920	Adipocytokine signaling pathway	Endocrine system	2
hsa0492	Glucagon signaling pathway	Endocrine system	1
hsa04932	Non-alcoholic fatty liver disease (NAFLD)	Endocrine and metabolic diseases	4
hsa04024	cAMP signaling pathway	Signal transduction	1
hsa04151	PI3K-Akt signaling pathway	Signal transduction	1
hsa04152	AMPK signaling pathway	Signal transduction	1
hsa04310	Wnt signaling pathway	Signal transduction	1
hsa04380	Osteoclast differentiation	Development	1
hsa04976	Bile secretion	Digestive system	1
hsa05016	Huntington’s disease	Neurodegenerative diseases	1
hsa05160	Hepatitis C	Infectious diseases	4
hsa05200	Pathways in cancer	Cancers	3
hsa05202	Transcriptional misregulation in cancer	Cancers	2
hsa05216	Thyroid cancer	Cancers	2
hsa05221	Acute myeloid leukemia	Cancers	1
hsa05222	Small cell lung cancer	Cancers	1
hsa05223	Non-small cell lung cancer	Cancers	1

## Supplementary Materials

Supplementary materials can be found at www.mdpi.com/1422-0067/17/12/2114/s1.
